# Comparison of Total Hemispherical Reflectance and Emittance Values Between Metformin Extended-Release Tablets Stored Under Ambient and Stress Conditions

**DOI:** 10.3390/s25030743

**Published:** 2025-01-26

**Authors:** Beata Sarecka-Hujar, Michał Meisner

**Affiliations:** 1 Department of Basic Biomedical Science, Faculty of Pharmaceutical Sciences in Sosnowiec, Medical University of Silesia in Katowice, 10 Jedności Str, 41-200 Sosnowiec, Poland; 2 Faculty of Pharmaceutical Sciences in Sosnowiec, Doctoral School of the Medical University of Silesia in Katowice, 41-200 Sosnowiec, Poland

**Keywords:** reflectance, emissivity, extended-release tablets, drug storage

## Abstract

Type 2 diabetes is a serious health problem worldwide. Metformin as the first-line drug in diabetes treatment mainly inhibits glucose production in the liver. Diabetes is often accompanied by other diseases, so patients may take many medications at the same time and have trouble controlling the therapy. This, in turn, may result in medications being stored in different, sometimes random places in the patient’s home where elevated temperatures or long-term exposure to solar radiation are possible. In this study, we aimed to analyze whether the total hemispherical reflectance and emittance values of metformin extended-release tablets would distinguish tablets stored correctly from those stored inconsistently with the manufacturer’s recommendations. Unexpired and expired extended-release tablets containing 750 mg metformin were tested. Unexpired tablets were analyzed in two ways i.e., 15 randomly selected tablets were stored as recommended (day 0), and the 15 next tablets in the blister were stored on a windowsill, where they were exposed to daylight for several hours during the day in mid-spring 2024 for 20 days (day 20). Total hemispherical reflectance (THR) was measured within seven spectral ranges from 335 nm to 2500 nm with a 410-Solar Reflectometer while emittance was analyzed within six spectral infrared ranges from 1500 nm to 21 microns with an ET 100 emissometer. The day 0 tablets showed the highest THR values in five spectral ranges from 400 to 1700 nm compared to expired and day 20 tablets. In the further infrared ranges, from 1.5 to 21 microns, unexpired tablets on day 0 had the lowest reflectance compared to day 20 tablets and expired tablets. This means that a greater amount of IR beam was absorbed by this type of tablet. Therefore, higher emittance was demonstrated by day 0 tablets than by other analyzed tablets. In addition, the emittance values for day 0 tablets decreased with increasing temperature. In conclusion, the storage of metformin extended-release tablets under unfavorable conditions may affect the physical structure of this drug form, which is manifested by changes in the reflectance and directional and hemispherical thermal emittance.

## 1. Introduction

The number of people suffering from type 2 diabetes is constantly increasing in all regions of the world, especially in the developed ones (e.g., Western Europe). In 2017, the rate of type 2 diabetes was about 6000 per 100,000 and is expected to increase to app. 7000 per 100,000 by 2030 [1]. More than 1 million people die each year from diabetes, making it the eighth most common cause of death [2]. In treating type 2 diabetes mellitus, metformin is the first-line drug showing pleiotropic effects on glucose metabolism. This effect is mainly due to the inhibition of glucose production in the liver, but the exact mechanism of action of metformin is not fully understood [3]. The typical diabetes treatment regimen involves taking metformin with meals (3–4 500 mg tablets or 2–3 850 mg tablets for a total daily dose).

Diabetes can affect more than 19% of people over 65 years of age, and according to the literature, more than 80% of these people are also affected by other chronic diseases. This increases the likelihood of polypharmacy [4], with up to 40% of patients over 65 estimated to use five or more prescription drugs. This carries an increased risk of difficulties in achieving therapeutic goals, due to non-adherence to prescriptions, incorrect storage of medicines, or storage beyond the expiration date [5]. A significant proportion of older single people who regularly use five or more medicines lack the knowledge and capacity to manage their medicines [6].

Data indicate that patients, especially the elderly, often do not pay attention to the storage conditions of medications and store them contrary to the manufacturer’s recommendations [7,8]. Thus, medicines can be temporarily stored in some places of the house where elevated temperatures or long-term exposure to solar radiation are possible. Photodegradation, induced by solar light, is a process of changes in a medicinal product’s chemical and physical composition. It can occur at any stage of a drug’s production, distribution, and storage which may ultimately lead to loss or transformation of the active ingredient, reduced efficacy, or the formation of new adventitious products [9].

Technologies that can be used for drug development and control are constantly evolving. Recently, we proposed using new analytical methods of hemispherical directional reflectance, microtomography, and hyperspectral imaging to analyze solid oral pharmaceutical preparations stored in various adverse conditions [10,11]. We proposed these methods as they are fast and non-destructive and can be easily used for screening pharmaceutical preparations in any test conditions. In addition, as the data show, these techniques can distinguish counterfeit from original drugs [12] and expired from unexpired ones [10,11]. In addition to hemispherical directional reflectance, the data on the emissivity of tablets can provide new data on drugs. Emissivity is defined as the ability of a material to emit energy. This parameter depends on several factors including the properties of the material surface and the surface conditions. It is also influenced by temperature and the wavelength of the radiation [13,14]. The emissivity value ranges from 0 (for a perfectly reflective surface) to 1 (for a perfectly emissive surface). It is the ratio of the radiation emitted by a surface to that emitted by a blackbody when their temperature is equal. Like emissivity, reflectance is described as an ability of a material. In this case, however, we are considering the ratio of incident radiation power to reflected radiation power. Emissivity and reflectivity are regarded as features of surface materials and their roughness (microscopic structure) but not their overall curvature [15].

The present study aimed to analyze the values of total hemispherical reflectance and emittance of metformin extended-release tablets within wide spectral ranges in ambient conditions and after exposure to sunlight falling on the drug through the window. We assumed that these parameters would be able to distinguish tablets stored correctly from those stored in a manner inconsistent with the manufacturers’ recommendations.

## 2. Materials and Methods

### 2.1. Model Tablets

Two types of extended-release tablets containing 750 mg of metformin from the same commercial preparation available on the Polish market were studied, i.e., (1) unexpired tablets (*n* = 30, randomly selected out of 60, expiration date May 2027) and (2) expired tablets (*n* = 15, randomly selected out of 60, stored according to manufacturer’s recommendations; expiration date July 2022).

**Figure 1:**
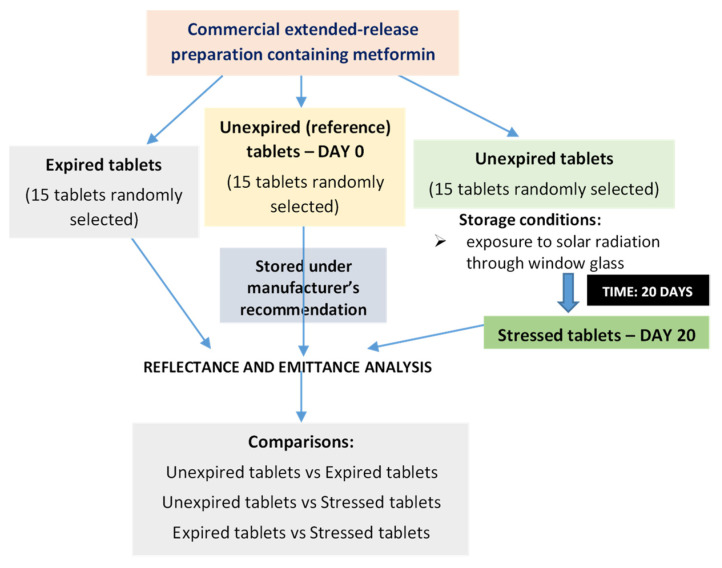
Diagram of the course and conditions of the experiment.

The unexpired tablets were analyzed in two ways: the first was without any intervention, i.e., tablets which were stored continuously before the experiment according to the manufacturer’s recommendations (*n* = 15, served as reference, DAY 0), and the second way was storage for 20 days under stress conditions similar to those that patients might generate at home (*n* = 15, stressed tablets, DAY 20). To create these, a randomly selected blister of extended-release metformin tablets from the main cardboard packaging was placed on a windowsill in our laboratory and therefore exposed to daylight through the window glass for several hours during the day in mid-spring 2024. Figure 1 shows a diagram of the course and conditions of the experiment.

### 2.2. Reflectance and Emittance Measurements

Total hemispherical reflectance (THR) was measured within 7 spectral ranges with a 410-Solar Reflectometer (Surface Optics Corporation, San Diego, CA, USA). The specific spectral ranges in which the numerical THR values were extracted were 335–380 nm, 400–540 nm, 480–600 nm, 590–720 nm, 700–1100 nm, 1000–1700 nm, and 1700–2500 nm for a beam at an angle of 20°. THR values were obtained for conditions corresponding to a solar zenith angle of 48° (air mass 1.5). Before measurement, the device was calibrated using the mirror and diffuse coupons provided by the manufacturer.

In turn, the emittance of the tablets was measured using an ET 100 emissometer (Surface Optics Corporation, San Diego, CA, USA). The device measures directional reflectance in the 6 thermal infrared spectral ranges from 1.5 to 21 microns. The specific ranges were 1.5–2.0, 2.0–3.5, 3.0–4.0, 4.0–5.0, 5.0–10.5, and 10.5–21 microns at two incidence angles—near normal 20° and near grazing 60°. Before measurement, calibration was performed with a specular gold coupon. Based on the values obtained, (1) directional thermal emissivity (DTE) at 20° and 60°, and (2) hemispherical thermal emissivity (HTE) were assessed for the unexpired, expired, and stressed tablets with metformin. In addition, the values of DTE and HTE were analyzed in 9 simulating temperatures from 300 K to 1200 K for the unexpired tablets.

The analyzed parameters of each analyzed tablet were assessed three times during a single measurement.

### 2.3. Statistical Analyses

The statistical evaluation of data was performed with Statistica 13 (StatSoft, Tulsa, OK, USA) and Microsoft Excel 2019 (Office 365, Microsoft Corporation, Redmond, WA, USA). We presented the THR and emittance values as mean ± standard deviation (M ± SD). With the Shapiro–Wilk W test, the normality of data distributions was assessed. The comparison of the variables between the two groups (i.e., unexpired tablets vs. expired tablets, unexpired tablets vs. stressed tablets, and expired tablets vs. stressed tablets) was performed with parametric tests (Student’s *t*-test) or non-parametric tests (Mann–Whitney U test) based on the distribution. Similarly, more than two groups of tablets were compared using ANOVA with repeated measures or the Kruskal–Wallis test. In the case of significant results from the comparisons, post hoc analyses were performed. A statistical significance was considered when a *p*-value was less than 0.05.

## 3. Results

### 3.1. Characteristics of the Tablets

The tablets were white to off-white, oblong, and biconvex. One extended-release tablet contained 750 mg of metformin hydrochloride, equivalent to 585 mg of metformin. In the tablets tested, magnesium stearate, carmellose sodium, and hypromellose 100,000 cPS were used as excipients.

### 3.2. Total Hemispherical Reflectance Analysis

**Figure 2:**
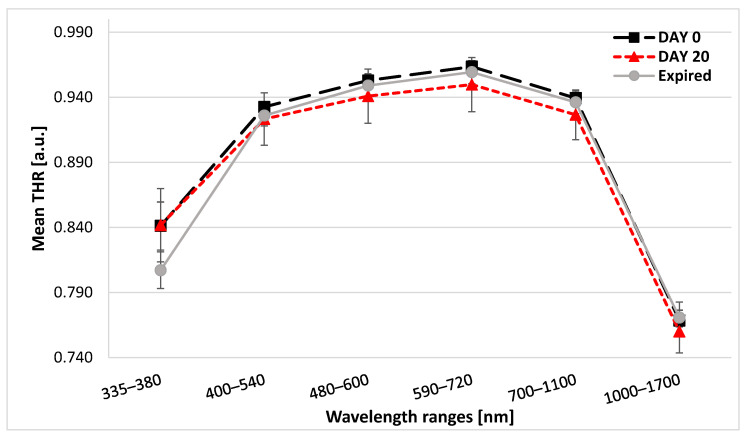
Graphs presenting mean values of THR for the analyzed types of extended-release tablets with metformin. The standard deviation bars are demonstrated in the chart. Data for the 1700–2500 nm range are not seen. THR—total hemispherical reflectance.

During the 20 days of storage on the windowsill, the blister changed its color from silver to light brown. Within the spectral range of 335–380 nm, expired tablets had the lowest mean values of THR while tablets on day 0 and day 20 had comparable THR. For the remaining wavelength ranges, the highest mean value of THR was demonstrated for the 590–720 nm range (Figure 2). In Figure 2, data for 1700–2500 nm are not seen as the THR values were the lowest (day 0: 0.422 ± 0.009; day 20: 0.422 ± 0.013, expired: 0.431 ± 0.012) for this range and no differences were visible for the remaining ranges.

Significant differences in THR values between the analyzed types of tablets were observed in all spectral ranges. In the post hoc analysis, in the range of 335–380 nm, the THR values differed significantly between day 0 vs. expired tablets and day 20 vs. expired tablets (*p* < 0.001). In the range of 400–540 nm, the only statistical difference was found between day 0 vs. expired tablets (*p* < 0.001). In turn, for 480–600 nm, day 20 tablets had significantly lower THR values compared to day 0 tablets (*p* = 0.014). Within the ranges of 590–700 nm and 700–1100 nm, the THR values for tablets on day 20 were lower than THR on day 0 (*p* < 0.001 each) and THR for expired tablets (*p* = 0.028 and *p* = 0.018, respectively). For the 1000–1700 nm band, the THR values for expired tablets were greater than THR for day 20 tablets (*p* = 0.007). On the contrary, in the 1700–2500 nm range, the THR values for expired tablets were significantly higher compared to day 0 tablets and day 20 tablets (*p* = 0.005 and *p* = 0.039, respectively) (Figure 2).

For infrared bands from 1.5 to 21.0 microns, the DHR values at both the 20° and 60° angles were evaluated. The DHR values at 20° differed significantly between day 0 and day 20 tablets in each spectral range (*p* < 0.001 each). In turn, in the 3.0–4.0 and 5.0–10.5 micron bands, the values of DHR at 20° were comparable between the day 0 and expired tablets. On the other hand, DHR was similar in all spectral ranges except for the 3.0–4.0 micron range for both the day 20 and expired tablets (Table 1).

When the DHR values were measured at a 60° angle, the differences between the tablets for all spectral bands except for the range of 5.0–10.5 microns were observed (Table 2). DHR at 60° had higher values than at 20°.

### 3.3. Emittance Analysis

**Figure 3:**
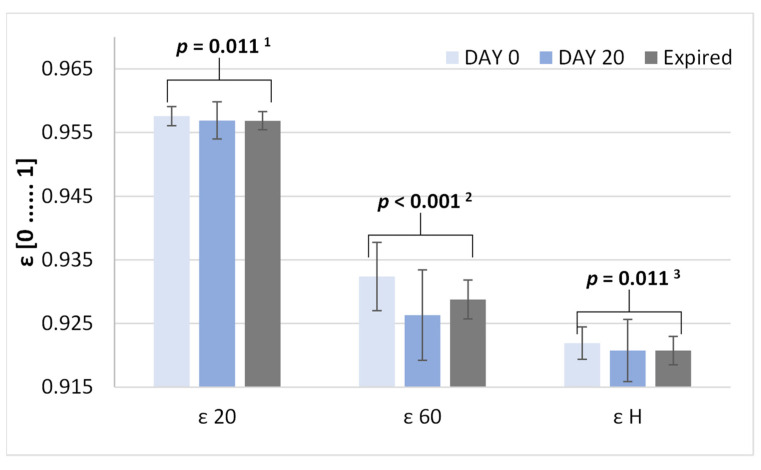
Comparison of mean ε 20, ε 60, and ε H between the tested extended-release tablets with metformin. The standard deviation bars are presented in the chart. Post hoc analysis: ^1^ day 0 vs. expired *p* = 0.022, day 0 vs. day 20 *p* = 0.050; ^2^ day 0 vs. expired *p* = 0.033, day 0 vs. day 20 *p* < 0.001; ^3^ day 0 vs. expired *p* = 0.027, day 0 vs. day 20 *p* = 0.036.

The analysis of emittance (ε 20, ε 60, and ε H) demonstrated differences between the tablets. Day 0 tablets had higher values of ε 20 compared to expired and day 20 tablets, but the ε 20 of expired and day 20 tablets was similar (Figure 3). In the case of ε 60, still, the day 0 tablets had the highest ε 60, while the ε 60 of day 20 tablets was the lowest. Also, the ε H parameter was the highest for the day 0 tablets. In turn, there was no difference in the emittance ε H of tablets on day 20 and the emittance of expired tablets (Figure 3).

**Figure 4:**
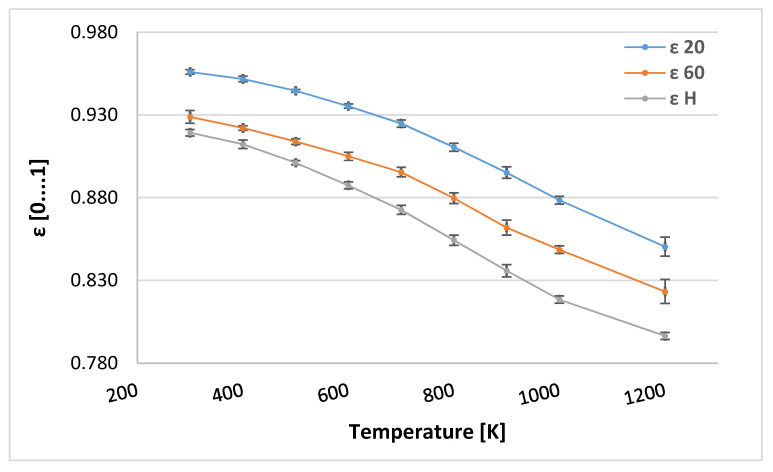
Mean values ε 20, ε 60, and ε H of the tested extended-release tablets with metformin in increasing temperature. The standard deviation bars are presented in the chart.

In addition, the DTE at 20 °C, DTE at 60 °C, and HTE values for day 0 tablets were evaluated as a function of temperature [K], showing a decrease with increasing temperature (Figure 4).

## 4. Discussion

In our study, the unexpired tablets on day 0, which were stored in the standard conditions (ambient temperature, away from the sunlight, and with relative humidity below 65%) before the experiment, showed the highest THR values in five spectral ranges from 400 to 1700 nm compared to the expired and stressed tablets. High THR values mean that such an amount of light reflects from the object when the rest of the light is transmitted/absorbed through/by the object. In the further infrared ranges, from 1.5 to 21 microns, the unexpired tablets on day 0 had the lowest reflectance compared to the day 20 tablets and expired tablets. This means that a greater amount of IR beam was absorbed by this type of tablet. Therefore, higher emittance was demonstrated by the unexpired tablets than by the other analyzed tablets. According to the principle of thermal emission, the emissivity of an object is equal to the absorptivity for a given frequency, direction, and polarization [16]. In our previous research, effervescent tablets with paracetamol and vitamin C stored under UV radiation showed higher emissivity than unexpired tablets kept in proper conditions [17], which may result from the fact that the surface of the effervescent tablets tended to be rougher in unfavorable conditions. In the study by Wen and Mudawar [18], a trend of decreasing reflectivity and therefore increasing emissivity was observed for rough aluminum alloy surfaces. In another study by the same group of researchers, for polished aluminum alloy, no trend of increasing emissivity with increasing temperature was observed for metal surfaces in the infrared range [19]. It was found that for most of the aluminum alloys tested, emissivity decreased between 600 and 700 K and increased between 700 and 800 K, while for the commercially pure aluminum, emissivity decreased with increasing temperature. In our study, the emissivity of the unexpired tablets decreased with temperature.

Hypromellose (HPMC) is the most commonly used substance to form swellable and soluble matrices [20]. It is a biopolymer derived from cellulose and has hydroxypropyl and methyl substituents in its structure. It exhibits good binding to drug molecules, usually through hydrogen bonding or weak Van der Waals forces, so that the drug molecules acquire a crystalline shape. The final properties of the polymer depend on the other components of the formulation [21]. Temperature affects the hydration of HPMCs, an increase in temperature results in a loss of hydration, which first translates into a decrease in relative viscosity, with further loss of water resulting in interactions between the polymer substituents [20]. Our results showed a decrease in the emissivity and reflectance of the tablets after UV exposure; this may be related to a change in the structure of the hypromellose-containing envelope. In a study conducted on nitrendipine tablets, the authors noted differences in the control of drug release following UV exposure [22]. As in our case, the envelope of the tablets tested consisted of hypromellose. The authors suggested that these changes may cause a loss of the desired therapeutic effect [22].

Although excipients are not expected to have any therapeutic effect, they have an important influence on the proper functioning of the finished drug formulation. Their function is to regulate the chemical and physical properties of the prepared medicine. Available studies show that they have a significant effect on maintaining the stability of the active substance. Unfortunately, the lack of appropriate regulations for stability and degradation testing of excipients is a barrier to the production of more efficient, stable, and safe medicines [23]. It is very easy to find data in the literature on the mechanisms by which excipients stabilize active substances, among them control of the water content of the drug formulation, change in pH, or acting as acid–base catalysts [24]. Exploring these mechanisms has allowed for an understanding of the interactions that can occur between API and excipients that directly translate into reduced drug stability. The implications of interactions between excipients were also recognized. A thorough understanding of these issues, as well as investigating the stability of excipients themselves under different conditions, will allow for a more accurate determination of the shelf life of finished formulations [24]. In this study, the effect of excipients on both the physical and chemical stability of levothyroxine sodium pentahydrate was assessed. The results showed that hygroscopic and acidic excipients accelerate its degradation [25]. Also, a study conducted by Gumieniczek et al. [26] on three antihistamines containing diphenhydramine, azelastine, and bepotastine in their composition confirmed the adverse effect of excipients on the stability of the finished preparation. Citric acid and polyvinyl alcohol showed interactions with the test active substances during exposure to UV radiation and elevated temperature. It was specified that citric acid decreased stability more under elevated temperature conditions while polyvinyl alcohol decreased stability under UV-VIS exposure [26]. However, another study by Parmar et al. [27] focused on the stability of a formulation containing lercanidipine and found that this substance was compatible with common excipients. Furthermore, moisture content and temperature were found to have the greatest effect on the degradation rate [27].

This study has some limitations. First, we did not provide information on the level of sunlight and temperature on the windowsill during the experiment because we focused only on storing the drug on the windowsill. In further studies, we plan to compare the values of the tested parameters of solid oral pharmaceutical preparations stored at the same time but in different seasons. Second, based on the results of this study, it is difficult to indicate possible modifications of the substances contained in the tablets in order to improve the formulation. Further studies are necessary on the analysis of single excipients, as well as mixtures of active substances with excipients, in order to propose substances with the highest properties of reflecting radiation, even after exposure to sunlight.

Since we are not able to determine whether the changes we observe occur in the active substance, the excipient, or perhaps in the physical properties of the drug form, it is important to focus on the entire drug form in the context of considering its stability because the multitude of mechanisms occurring during the storage of the drug product means that analysis of only one parameter may not be reliable. However, the results we obtained undoubtedly prove that the solid oral form of the drug changes during storage in unfavorable conditions, and the change can be effectively confirmed by the methods of hemispherical directional reflectance and emissivity.

## 5. Conclusions

The storage of metformin extended-release tablets under unfavorable conditions may affect the physical structure of this drug form, which is manifested by changes in the reflectance and directional and hemispherical thermal emittance. The THR value, in the range of visible and infrared light (from 700–2500 nm), is the lowest for day 20 tablets stored on a windowsill after exposure to sunlight falling on the tablets through the window glass. In the range of UV light, i.e., 335–380 nm, expired tablets had the lowest THR value. A lower THR value means that a greater amount of radiation was absorbed by this type of tablet. In turn, in the further infrared ranges, from 1.5 to 21 microns, unexpired tablets on day 0 had the lowest reflectance compared to day 20 tablets and expired tablets. Tablets after irradiation with sunlight showed lower emittance than other types of tablets.

## Figures and Tables

**Table 1:** The juxtaposition of mean directional reflectance values for day 0 (unexpired), expired, and day 20 (stressed) metformin tablets for a 20° angle.

Type of the Tablets	DHR Values for 20° Angle			*p*
IR Bands	Unexpired Day 0	Stressed Day 20	Expired	
1.5–2.0 microns	0.470 ± 0.012	0.477 ± 0.014	0.473 ± 0.016	**<0.001**
2.0–3.5 microns	0.236 ± 0.006	0.243 ± 0.009	0.242 ± 0.010	**<0.001**
3.0–4.0 microns	0.042 ± 0.007	0.046 ± 0.007	0.042 ± 0.008	**<0.001**
4.0–5.0 microns	0.108 ± 0.004	0.114 ± 0.006	0.113 ± 0.006	**<0.001**
5.0–10.5 microns	0.046 ± 0.002	0.047 ± 0.001	0.046 ± 0.001	**<0.001**
10.5–21.0 microns	0.038 ± 0.003	0.040 ± 0.002	0.040 ± 0.002	**<0.001**

DHR—directional hemispherical reflectance; IR—infrared. Significant differences are in bold.

**Table 2:** The juxtaposition of mean directional reflectance values for unexpired, expired, and stressed metformin tablets for a 60° angle.

Type of the Tablets	DHR Values for 20° Angle			*p*
IR Bands	Unexpired Day 0	Stressed Day 20	Expired	
1.5–2.0 microns	0.470 ± 0.012	0.477 ± 0.014	0.473 ± 0.016	**<0.001**
2.0–3.5 microns	0.236 ± 0.006	0.243 ± 0.009	0.242 ± 0.010	**<0.001**
3.0–4.0 microns	0.042 ± 0.007	0.046 ± 0.007	0.042 ± 0.008	**<0.001**
4.0–5.0 microns	0.108 ± 0.004	0.114 ± 0.006	0.113 ± 0.006	**<0.001**
5.0–10.5 microns	0.046 ± 0.002	0.047 ± 0.001	0.046 ± 0.001	**<0.001**
10.5–21.0 microns	0.038 ± 0.003	0.040 ± 0.002	0.040 ± 0.002	**<0.001**

DHR—directional hemispherical reflectance; IR—infrared. Significant differences are in bold.

## Data Availability

The data presented in this study are available on request from the Department of Basic Biomedical Science, Faculty of Pharmaceutical Sciences in Sosnowiec, Medical University of Silesia in Katowice (Poland). The data are not publicly available due to privacy restrictions.
